# Contemporary Patterns of End-of-Life Care Among Medicare Beneficiaries With Advanced Cancer

**DOI:** 10.1001/jamahealthforum.2024.5436

**Published:** 2025-02-21

**Authors:** Youngmin Kwon, Xin Hu, Kewei Sylvia Shi, Jingxuan Zhao, Changchuan Jiang, Qinjin Fan, Xuesong Han, Zhiyuan Zheng, Joan L. Warren, K. Robin Yabroff

**Affiliations:** 1 Department of Health Policy, Vanderbilt University Medical Center, Nashville, Tennessee; 2 American Cancer Society, Atlanta, Georgia; 3 Division of Health Services Research, Outcomes, and Policy, Department of Radiation Oncology, Emory University School of Medicine, Atlanta, Georgia; 4 Department of Medicine, UT Southwestern Medical Center, Dallas, Texas

## Abstract

**Importance:**

Considerable efforts have been dedicated to improving the quality of end-of-life care among patients with advanced cancer in the past decade. Whether the quality has shifted in response to these efforts remains unknown.

**Objective:**

To examine contemporary patterns of end-of-life care among patients with advanced cancer.

**Design, Setting, and Participants:**

This retrospective cohort study used a recent linkage of Surveillance, Epidemiology, and End Results and Medicare data to characterize patterns of end-of-life care. The cohort included fee-for-service Medicare decedents aged 66 years or older who were originally diagnosed with distant-stage breast, prostate, pancreatic, or lung cancers and died between 2014 and 2019. Analyses were conducted between June 1, 2023, and July 31, 2024.

**Main Outcomes and Measures:**

Outcomes included monthly use of acute care, systemic therapy, and supportive care (ie, palliative and hospice care and advanced care planning) in the last 6 months of life. Additionally, a claims-based indicator was evaluated of potentially aggressive care in the last 30 days of life, defined as experiencing more than 1 acute care visit, in-hospital mortality, late receipt of systemic therapy, or hospice entry.

**Results:**

The study included 33 744 Medicare decedents with advanced cancer (mean [SD] age, 75.7 [6.9] years; 52.1% male). From 6 months before death to month of death, there was an increase in the mean (SE) number of acute care visits (from 14.0 [0.5] to 46.2 [0.5] per 100 person-months), hospice use (from 6.6 [0.4] to 73.5 [0.5] per 100 person-months), palliative care (from 2.6 [0.2] to 26.1 [0.6] per 100 person-months), and advanced care planning (from 1.7 [0.6] to 12.8 [1.1] per 100 person-months). Overall, 45.0% of decedents experienced any indicator of potentially aggressive care.

**Conclusions and Relevance:**

This study found persistent patterns of potentially aggressive care, but low uptake of supportive care, among Medicare decedents with advanced cancer. A multifaceted approach targeting patient-, physician-, and system-level factors associated with potentially aggressive care is imperative for improving quality of care at the end of life.

## Key Points

**Question:**

What are contemporary patterns of end-of-life care in patients with advanced cancer in the US?

**Findings:**

In this retrospective cohort study of 33 744 fee-for-service Medicare decedents aged 66 years or older with advanced cancer from 2014 to 2019, 45.0% experienced a claims-based indicator of potentially aggressive care, driven by an increase in acute care and low receipt of supportive care (palliative and hospice care and advanced care planning) near death.

**Meaning:**

Despite major focus on optimizing the quality of end-of-life care, this study revealed potentially aggressive care of advanced cancer, at the expense of supportive care, in oncology.

## Teaser

This cohort study examines the current patterns of end-of-life care among older, fee-for-service Medicare decedents with advanced cancer.

## Introduction

Cancer is the second leading cause of death in the US, with more than 611 720 cancer deaths in 2024.^1^ For patients with advanced cancers, the goal of care at end of life (EOL) is to maximize remaining quality of life. Optimal EOL care emphasizes symptom management and palliation, including hospice for patients ineligible for care with curative intent.^2,3^ Contrary to this goal, historical evidence has shown that patients with advanced cancer often receive potentially aggressive care at EOL, such as late initiation of systemic therapies and/or acute care use.^2,4,5,6^

Clinicians and professional societies have dedicated considerable efforts to improve the quality of EOL care in oncology in the past decade. From 2009 to 2017, new quality measures and guidelines on EOL care were established by the National Quality Forum and American Society of Clinical Oncology (ASCO), recommending early integration of palliative care and discontinuation of life-extending therapies.^7,8,9^ These efforts are situated within the Choosing Wisely initiative, a large campaign aimed at raising awareness of the harms of aggressive medical care.^10,11,12^ Moreover, the Centers for Medicare & Medicaid Services began reimbursing advanced care planning (ACP) conversations in 2016 to further the goals of high-quality, patient-centered EOL care.^13^ Whether EOL care patterns shifted in response to these efforts is unknown because much contemporary evidence on this topic preceded these efforts.^2,5,6,14,15,16,17,18,19,20,21^

Therefore, we conducted a retrospective cohort study of EOL care patterns in older, fee-for-service (FFS) Medicare beneficiaries dying of cancer between 2014 and 2019, incorporating the most recent sample of Medicare decedents from the Surveillance, Epidemiology, and End Results (SEER)–Medicare data linkage (at the time of study initiation). We examined a comprehensive set of measures to investigate care quality, including receipt of acute care, systemic therapies, palliative care (PC) and hospice care (HC), and ACP. We focus on patients originally diagnosed with distant-stage female breast, prostate, pancreatic, or lung cancer, for whom the quality of EOL care is a salient concern given poor prognosis at diagnosis.

## Methods

### Data Source

The SEER program is composed of population-based cancer registries funded by the National Cancer Institute to collect information about persons diagnosed with incident cancers in defined geographic areas. The registries included in this analysis represent approximately 26% of the US population^22^ and report each patient’s type of cancer, stage, month and year of diagnosis, cause of death, and other demographics. Persons included in the SEER data are linked to administrative Medicare data. Medicare information used for this study included exact date of death and health claims for hospitalizations (Medicare Provider Analysis and Review), physician services (Carrier), outpatient services, HC, and prescription drugs covered under the pharmacy benefit (Part D). Medicare claims include dates of service and codes for specific diagnoses and procedures, which we used to identify EOL care measures. This study has been exempted from review and the need for informed consent by the Morehouse School of Medicine Institutional Review Board because it analyzed deidentified data. We followed the Strengthening the Reporting of Observational Studies in Epidemiology (STROBE) reporting guideline.

### Study Population

We identified Medicare beneficiaries 66 years or older who were initially diagnosed with distant-stage female breast, prostate, pancreas, or lung cancer and died of cancer between 2014 and 2019 (eTable 1 in Supplement 1). To ensure representativeness, we included only patients with primary histologic types for breast, prostate, pancreatic, non–small cell lung (NSCL), and small cell lung (SCL) cancers using *International Classification of Diseases for Oncology, Third Edition* (*ICD-O-3*) codes (eTable 2 in Supplement 1). To identify those who died of cancer, we used the cause-of-death variable in SEER, populated from death certificates, which is a validated method for ascertaining cancer-specific deaths.^23,24,25^ We further required a single cancer diagnosis, survival for 30 days or more after diagnosis, and continuous enrollment in FFS Medicare Part A and B coverage in the 6 months before diagnosis and the last 6 months of life. For analysis of systemic therapies, we additionally required continuous Part D enrollment to capture both infused and oral regimens. For analysis of ACP, we restricted the sample to decedents who died in or after 2016 when ACP became a Medicare-billable service.

### EOL Care Use Measures

To describe patterns of EOL care, we first evaluated monthly use of health care services in the last 6 months of life, including acute care (ie, hospitalization or emergency department [ED] visits), receipt of systemic therapies, and supportive care (ie, PC, HC, and ACP) (eTable 3 in Supplement 1). For hospital admissions, we characterized unique hospitalizations based on admission and discharge dates. We identified ED visits following the Research Data Assistance Center algorithm.^26^ Systemic therapies included infused and oral therapies. When examining hospice use, we assumed that patients were likely to remain enrolled in hospice on entry and counted the earliest available hospice claim per patient. Because there are no specific procedure codes for PC,^27^ we used the specialty code for PC (Hospice and Palliative Care) on the outpatient and physician carrier claims and *International Classification of Diseases, Ninth Revision* (*ICD-9*) and *International Statistical Classification of Diseases and Related Health Problems, Tenth Revision* (*ICD-10*) diagnosis codes for PC encounters on inpatient, outpatient, and carrier claims. To distinguish PC provided in vs out of hospice settings, we removed PC claims for patients who were already in hospice or entered hospice within 7 days of any PC claim identified using the provider specialty code. Then, we assessed claims-based indicators of potentially aggressive care in the last 30 days of life^5,6,14^: more than 1 ED visit or hospitalization, any intensive care unit admissions, in-hospital death, hospice enrollment less than 3 days before death, any systemic therapy 14 days or less before death, and a composite outcome for having at least 1 of these indicators.

### Covariates

From the SEER data, we characterized demographic variables (sex, age at diagnosis, race and ethnicity, marital status, year of death, and state of residence), cancer type, duration of survival, and other area-level variables (Yost Index of socioeconomic status quintile^28,29,30^ and rurality). Race and ethnicity information was abstracted from medical records using standardized fields by registrars, but there may be misclassification of race and ethnicity by the reporting health care facilities and clinicians submitting such records. We collected data on race and ethnicity to describe the representativeness of the sample. We combined race and ethnicity variables into the following groups: Hispanic, non-Hispanic Black, non-Hispanic White, and other or unknown (including patients with non-Hispanic ethnicity and 1 or more of the following races: Alaska Native, Aleutian, American Indian, or Eskimo; Asian Indian or Pakistani; Chamorran; Chinese; Fiji Islander; Filipino; Guamanian; Hawaiian; Hmong; Japanese; Kampuchean; Korean; Laotian; Melanesian; Micronesian; New Guinean; Polynesian; Samoan; Tahitian; Thai; Tongan; Vietnamese; other Asian or Pacific Islanders; or other or unknown race). Rurality was characterized at the county level using the Rural-Urban Continuum Codes based on the 2010 US Census.^28^ We determined dual enrollment in Medicaid in the last 6 months of life and National Cancer Institute comorbidity index, calculated using all claims within 6 months before diagnosis.^31,32^

### Statistical Analysis

Analyses were conducted between June 1, 2023, and July 31, 2024. For monthly use outcomes in the last 6 months of life, we report rates of use per person-month (PM) when a patient was alive after diagnosis (to account for differences in survival after diagnosis).^33^ For each 30-day interval, we aggregated days alive after diagnosis to PM, the denominator. The numerator included the number of patients receiving service within a given PM; rates are the number of patients treated per 100 PM. To account for potential differences in care patterns by clinical characteristics, we also report rates by cancer type. In our analysis of claims-based indicators of potentially aggressive care, we report unadjusted mean outcomes (pooled across all years) as well as trends by year of death. We also conducted multivariate analyses that estimated adjusted probabilities of receiving potentially aggressive EOL care and any PC (using linear regression models) and mean duration in hospice in the last 30 days of life (estimated via a 2-part model of mean days in hospice, conditional on hospice enrollment, with a Poisson distribution^34^). Lastly, we examined annual receipt of PC to investigate how the recent ASCO recommendation to incorporate PC in usual oncology care (published in 2016^8^) may have influenced uptake of PC. We used an α of .05 to assess statistical significance.

## Results

### Sample Characteristics

We included 33 744 FFS Medicare beneficiaries aged 66 years or older who died of breast (n = 2086), prostate (n = 3239), pancreatic (n = 5595), NSCL (n = 18 150), and SCL (n = 4674) cancers (Table 1). Overall, 47.9% were female and 52.1% were male; mean (SD) age was 75.7 (6.9) years; and 5.0% were Hispanic, 8.9% were non-Hispanic Black, 80.5% were non-Hispanic White, and 5.6% were of other or unknown race and ethnicity. A total of 23 427 beneficiaries (69.4%) were continuously enrolled in Part D. Nearly all observed characteristics differed across cancer type; notably, survival following diagnosis was longer among patients with breast or prostate cancer compared with patients with pancreatic, NSCL, or SCL cancers. Sample characteristics were similar by year of death (eTable 4 in Supplement 1). Most patients (93.6%) were diagnosed between 2013 and 2019 (eFigure 1 in Supplement 1).

**Table 1.** Characteristics of Older, Fee-for-Service Medicare Beneficiaries Dying of Advanced Cancer Between 2014 and 2019 in SEER-Medicare, Overall and by Cancer Type

Characteristic^a^	No. (%) of participants					
	Overall (N = 33 744)	Breast cancer (n = 2086)	Prostate cancer (n = 3239)	Pancreatic cancer (n = 5595)	NSCL cancer (n = 18 150)	SCL cancer (n = 4674)
Sex						
Male	17 588 (52.1)	0	3239 (100)	2696 (48.2)	9379 (51.7)	2274 (48.7)
Female	16 156 (47.9)	2086 (100)	0	2899 (51.8)	8771 (48.3)	2400 (51.3)
Age at diagnosis, y						
66-69	7381 (21.9)	445 (21.3)	569 (17.6)	1324 (23.7)	3799 (20.9)	1244 (26.6)
70-74	9045 (26.8)	513 (24.6)	720 (22.2)	1492 (26.7)	4817 (26.5)	1503 (32.2)
75-79	7538 (22.3)	452 (21.7)	667 (20.6)	1256 (22.4)	4124 (22.7)	1039 (22.2)
80-85	9780 (29)	676 (32.4)	1283 (39.6)	1523 (27.2)	5410 (29.8)	888 (19)
Race and ethnicity^b^						
Hispanic	1690 (5)	95 (4.6)	206 (6.4)	376 (6.7)	845 (4.7)	168 (3.6)
Non-Hispanic Black	3010 (8.9)	246 (11.8)	364 (11.2)	485 (8.7)	1612 (8.9)	303 (6.5)
Non-Hispanic White	27 171 (80.5)	1661 (79.6)	2540 (78.4)	4450 (79.5)	14449 (79.6)	4071 (87.1)
Other or unknown	1873 (5.6)	84 (4.0)	129 (4.0)	284 (5.1)	1244 (6.9)	132 (2.8)
Marital status^c^						
Single	3614 (10.7)	267 (12.8)	410 (12.7)	513 (9.2)	1966 (10.8)	458 (9.8)
Married	17 029 (50.5)	688 (33.0)	1908 (58.9)	3199 (57.2)	8951 (49.3)	2283 (48.8)
Separated, divorced, widowed	11 664 (34.6)	1002 (48.0)	721 (22.3)	1675 (29.9)	6520 (35.9)	1746 (37.4)
Unknown	1437 (4.3)	129 (6.2)	200 (6.2)	208 (3.7)	713 (3.9)	187 (4)
Dually enrolled in Medicaid^d^						
Any dual enrollment	6623 (19.6)	448 (21.5)	643 (19.9)	806 (14.4)	3793 (20.9)	933 (20.0)
No dual enrollment	27 121 (80.4)	1638 (78.5)	2596 (80.1)	4789 (85.6)	14357 (79.1)	3741 (80.0)
Part D enrollment						
Continuously enrolled	23 427 (69.4)	1483 (71.1)	2141 (66.1)	3941 (70.4)	12620 (69.5)	3242 (69.4)
No continuous enrollment	10 317 (30.6)	603 (28.9)	1098 (33.9)	1654 (29.6)	5530 (30.5)	1432 (30.6)
Duration of survival, mo^e^						
<6	18 435 (54.6)	687 (32.9)	516 (15.9)	3872 (69.2)	10678 (58.8)	2682 (57.4)
6-18	8699 (25.8)	444 (21.3)	847 (26.2)	1297 (23.2)	4526 (24.9)	1585 (33.9)
≥18	6610 (19.6)	955 (45.8)	1876 (57.9)	426 (7.6)	2946 (16.2)	407 (8.7)
NCI comorbidities index^f^						
0	12 430 (36.8)	1136 (54.5)	1624 (50.1)	2078 (37.1)	6321 (34.8)	1271 (27.2)
0-1	14 978 (44.4)	692 (33.2)	1182 (36.5)	2604 (46.5)	8210 (45.2)	2290 (49)
1-2	5371 (15.9)	223 (10.7)	366 (11.3)	774 (13.8)	3079 (17)	929 (19.9)
>3	965 (2.9)	35 (1.7)	67 (2.1)	139 (2.5)	540 (3.0)	184 (3.9)
Year of death						
2014	5767 (17.1)	324 (15.5)	487 (15.0)	877 (15.7)	3255 (17.9)	824 (17.6)
2015	5708 (16.9)	327 (15.7)	466 (14.4)	863 (15.4)	3234 (17.8)	818 (17.5)
2016	5818 (17.2)	353 (16.9)	538 (16.6)	936 (16.7)	3178 (17.5)	813 (17.4)
2017	5757 (17.1)	383 (18.4)	568 (17.5)	1001 (17.9)	3016 (16.6)	789 (16.9)
2018	5562 (16.5)	337 (16.2)	619 (19.1)	1009 (18)	2842 (15.7)	755 (16.2)
2019	5132 (15.2)	362 (17.4)	561 (17.3)	909 (16.2)	2625 (14.5)	675 (14.4)
Yost Index of SES quintile^g^						
1 (Low SES)	6040 (17.9)	370 (17.7)	555 (17.1)	722 (12.9)	3334 (18.4)	1059 (22.7)
2	6019 (17.8)	350 (16.8)	497 (15.3)	847 (15.1)	3373 (18.6)	952 (20.4)
3	6335 (18.8)	419 (20.1)	604 (18.6)	1026 (18.3)	3396 (18.7)	890 (19.0)
4	6815 (20.2)	432 (20.7)	686 (21.2)	1207 (21.6)	3644 (20.1)	846 (18.1)
5 (High SES)	7853 (23.3)	470 (22.5)	836 (25.8)	1676 (30)	4037 (22.2)	834 (17.8)
Missing	682 (2.0)	45 (2.2)	61 (1.9)	117 (2.1)	366 (2.0)	93 (2.0)
Rurality^h^						
Metropolitan	27 542 (81.6)	1727 (82.8)	2652 (81.9)	4787 (85.6)	14787 (81.5)	3589 (76.8)
Urban	3985 (11.8)	245 (11.7)	383 (11.8)	528 (9.4)	2177 (12)	652 (13.9)
Rural	2127 (6.6)	114 (5.5)	204 (6.3)	280 (5.0)	1186 (6.5)	433 (9.3)
SEER cancer registry						
California	9972 (29.6)	580 (27.8)	1047 (32.3)	1847 (33)	5482 (30.2)	1016 (21.7)
Connecticut	1854 (5.5)	104 (5.0)	238 (7.3)	328 (5.9)	948 (5.2)	236 (5.0)
Georgia	4136 (12.3)	249 (11.9)	358 (11.1)	641 (11.5)	2199 (12.1)	689 (14.7)
Iowa	2378 (7.0)	114 (5.5)	279 (8.6)	359 (6.4)	1219 (6.7)	407 (8.7)
Kentucky	3087 (9.1)	176 (8.4)	188 (5.8)	298 (5.3)	1757 (9.7)	668 (14.3)
Louisiana	2172 (6.4)	144 (6.9)	176 (5.4)	351 (6.3)	1161 (6.4)	340 (7.3)
Detroit	2200 (6.5)	158 (7.6)	159 (4.9)	360 (6.4)	1209 (6.7)	314 (6.7)
New Jersey	4618 (13.7)	351 (16.8)	392 (12.1)	839 (15.0)	2466 (13.6)	570 (12.2)
New Mexico	663 (2.0)	37 (1.8)	102 (3.1)	105 (1.9)	333 (1.8)	86 (1.8)
Utah	584 (1.7)	50 (2.4)	90 (2.8)	128 (2.3)	255 (1.4)	61 (1.3)
Seattle	2080 (6.2)	123 (5.9)	210 (6.5)	339 (6.1)	1121 (6.2)	287 (6.1)

Abbreviations: NSCL, non–small cell lung; SCL, small cell lung; NCI, National Cancer Institute; SES, socioeconomic status; SEER, Surveillance, Epidemiology, and End Results.

^a^ We report characteristics of fee-for-service Medicare beneficiaries older than 65 years who died of stage IV or distant-stage breast, prostate, pancreas, NSCL, or SCL cancer between 2014 and 2019 in SEER-linked Medicare files.

^b^ Race and ethnicity information are abstracted from medical records using standardized fields by cancer registrars, but there may be misclassification of race and ethnicity by the reporting health care facilities and clinicians submitting such medical records. SEER provides separate variables for race and ethnicity, which we collapsed into the listed categories. The other category includes patients with non-Hispanic ethnicity and 1 or more of the following races: Alaska Native, Aleutian, American Indian, or Eskimo; Asian Indian or Pakistani; Chamorran; Chinese; Fiji Islander; Filipino; Guamanian; Hawaiian; Hmong; Japanese; Kampuchean; Korean; Laotian; Melanesian; Micronesian; New Guinean; Polynesian; Samoan; Tahitian; Thai; Tongan; Vietnamese; other Asian or Pacific Islanders; and other or unknown race.

^c^ Marital status at the time of diagnosis that are self-reported in medical records. SEER’s definition of common law marriage includes a couple living together and declaring themselves as married, not requiring any formal ceremony or a marriage license.

^d^ We identified dual enrollment using the Medicaid buy-in codes from the linked Medicare master beneficiary files, including both partial or full Medicaid coverage.

^e^ We calculated months from diagnosis to death using the death date in the linked Medicare files that provides the exact date of death as opposed to death month (as is the case in SEER); all cases in the sample had agreeing death date information in both SEER and the linked Medicare files.

^f^ The NCI comorbidity index is a modified version of the Charlson Comorbidity Index that excludes diagnoses of solid tumors, leukemias, and lymphoma. We calculated the comorbidities index using Medicare Part A and B claims occurring within 6 months before diagnosis.

^g^ We characterized the Yost Index of SES quintile variable provided by SEER, which is calculated at the US Census tract level using the 2013 to 2014 American Community Survey; the first (fifth) quintile is the US Census tract group with the lowest (highest) SES.

^h^ We categorized the US Census tract–level rurality based on the identification of urban and rural areas from the 2010 US Census; urban (rural) denotes US Census tracts with more than 50% share of the population living in an urban (rural) area.

### Trajectory of EOL Care

**Figure 1.**
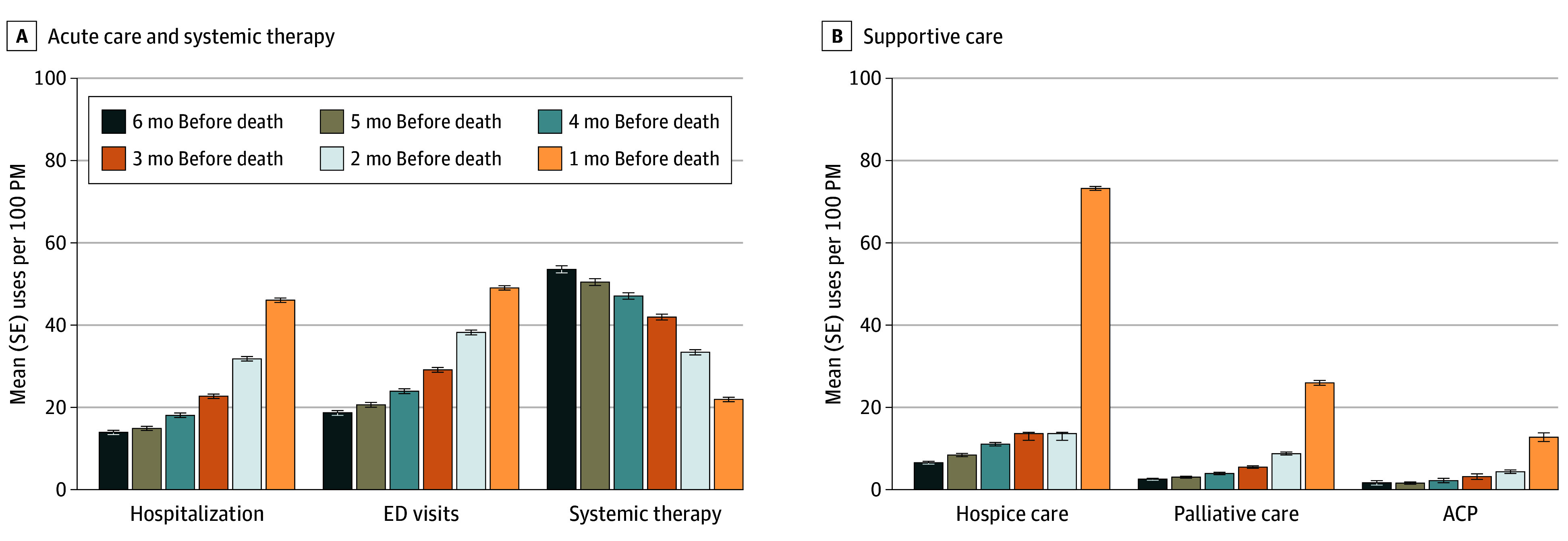
Monthly Rates of Health Care Use From 6 Months Before Death to the Month of Death for Each Service Type The monthly rate is calculated as the number of patients using health care services divided by the person-days when patients were alive and diagnosed with cancer converted to a person-months (PM) denominator (this accounts for the fact that not all patients survived at least 6 months). The denominator for systemic therapy excluded patients without continuous Part D enrollment in the last 6 months of life. Palliative care refers to supportive care provided outside of hospice. Advanced care planning (ACP) use was only measured for the sample dying at or after 2016. Error bars indicate SEs. ED indicates emergency department.

In the last 6 months of life, acute care use increased as patients approached death (Figure 1). The rate of hospitalization increased from a mean (SE) of 14.0 (0.5) per 100 PM at 6 months before death to 46.2 (0.5) per 100 PM in the month of death. The rate of ED visits increased from a mean (SE) of 18.7 (0.6) to 49.2 (0.5) per 100 PM, with most ED visits resulting in hospitalization. Conversely, receipt of systemic therapies decreased from a mean (SD) of 53.7 (0.9) to 22.0 (0.5) per 100 PM. These trajectories were generally consistent across cancer types, although patients with prostate cancer had lower rates of hospital admissions and receipt of systemic therapies, whereas patients with lung cancer had higher ED visit rates (eFigure 2 in Supplement 1). Supportive care trajectories had a similar pattern to acute care, where use spiked in the month of death but the use of PC and ACP remained low overall. Specifically, use of HC, PC, and ACP increased from a mean (SE) of 6.6 (0.4) to 73.5 (0.5) PM, 2.6 (0.2) to 26.1 (0.6) PM, and 1.7 (0.6) to 12.8 (1.1) per 100 PM, respectively. In addition, approximately one-quarter of the sample received any PC in last 6 months prior to death. Use of PC increased over time, from 21% in 2014 to 35% in 2019 (eFigure 3 in Supplement 1). Patients who were older, non-Hispanic White, residing in lower Yost Index of socioeconomic status quintiles, or had a longer survival duration were less likely to receive any PC in adjusted analyses (Table 2).

**Table 2.** Patient Characteristics Associated With Receipt of Any Palliative Care and Hospice Stays^a^

Variable	Probability of receiving palliative care in last 6 mo of life		Days in hospice in last 30 d of life	
	Adjusted marginal effect (95% CI)	*P* value	Adjusted marginal effect (95% CI)	*P* value
Cancer type				
NSCLC	1.0 [Reference]	NA	1.0 [Reference]	NA
Breast	1.07 (−1.20 to 3.34)	.35	−1.28 (−2.29 to 0.37)	.13
Prostate	1.47 (−0.51 to 3.44)	.15	1.84 (0.25 to 3.43)	.02
Pancreas	−1.5 (−2.9 to 0.0)	.04	2.30 (1.17 to 3.43)	<.001
SCLC	−0.18 (−1.72 to 1.37)	.82	−3.15 (−4.20 to −2.10)	<.001
Time from diagnosis to death, mo				
<6	1.0 [Reference]	NA	1.0 [Reference]	NA
6-18	−7.25 (−8.48 to −6.02)	<.001	15.64 (14.59 to 16.69)	<.001
≥18	−10.87 (−12.35 to −9.39)	<.001	21.14 (19.64 to 22.64)	<.001
Sex^b^				
Male	1.0 [Reference]	NA	1.0 [Reference]	NA
Female	1.40 (0.26 to 2.54)	.02	4.60 (3.72 to 5.48)	<.001
Age at death, y				
66-70	1.0 [Reference]	NA	1.0 [Reference]	NA
70-74	−1.96 (−3.42 to −0.49)	.009	1.67 (0.70 to 2.65)	.001
75-80	−4.39 (−5.92 to −2.85)	<.001	3.93 (2.86 to 5.00)	<.001
80-85	−9.59 (−11.07 to −8.11)	<.001	10.50 (9.34 to 11.67)	<.001
Race and ethnicity^b^^,^^c^				
Hispanic	3.67 (1.20 to 6.13)	.004	−3.08 (−4.79 to −1.37)	<.001
Non-Hispanic Black	8.31 (6.37 to 10.25)	<.001	−3.10 (−4.51 to −1.68)	<.001
Non-Hispanic White	1.0 [Reference]	NA	1.0 [Reference]	NA
Other or unknown	4.12 (1.75 to 6.50)	.001	−2.61 (−4.41 to −0.81)	.004
Marital status at diagnosis^b^				
Married	1.0 [Reference]	NA	1.0 [Reference]	NA
Unmarried	−0.85 (−1.97 to 0.27)	.14	3.16 (2.31 to 4.01)	<.001
Unknown	−5.13 (−7.72 to −2.55)	<.001	2.13 (0.11 to 4.16)	.04
Dually enrolled in Medicaid^d^				
No	1.0 [Reference]	NA	1.0 [Reference]	NA
Yes	0.56 (−0.86 to 1.99)	.44	3.87 (2.66 to 5.09)	<.001
NCI Comorbidity Index^d^^,^^e^				
0	1.0 [Reference]	NA	1.0 [Reference]	NA
0-1	0.54 (−0.61 to 1.69)	.36	1.10 (0.24 to 1.96)	.01
1-2	1.31 (−0.25 to 2.87)	.10	1.36 (0.15 to 2.56)	.03
>3	2.07 (−1.08 to 5.22)	.20	0.47 (−1.98 to 2.91)	.71
Yost Index of SES quintiles^b^^,^^f^				
1(Low SES)	1.0 [Reference]	NA	1.0 [Reference]	NA
2	0.91 (−0.85 to 2.67)	.31	−1.76 (−3.13 to −0.39)	.01
3	1.14 (−0.67 to 2.95)	.22	−2.13 (−3.52 to −0.74)	.003
4	2.12 (0.25 to 3.99)	.03	−1.09 (−2.54 to 0.36)	.14
5 (High SES)	4.86 (2.96 to 6.77)	<.001	−1.27 (−2.75 to 0.21)	.09
Unknown	4.56 (0.70 to 8.41)	.02	0.81 (−2.35 to 3.97)	.62
Rurality^b^^,^^g^				
Metropolitan	1.0 [Reference]	NA	1.0 [Reference]	NA
Urban	−6.33 (−8.07 to −4.58)	<.001	−1.46 (−2.75 to −0.18)	.03
Rural	−8.08 (−10.43 to −5.71)	<.001	−3.44 (−5.06 to −1.82)	<.001

Abbreviations: NA, not applicable; NCI, National Cancer Institute; NSCLC, non-small cell lung cancer; SCLC, small cell lung cancer; SES, socioeconomic status.

^a^ The table displays the adjusted marginal effects (95% CIs and *P* values) from a linear probability model that estimated the probability of receiving any palliative care in the last 6 months of life and a 2-part model with a Poisson distribution that estimated mean days spent in hospice in last 30 days, respectively. We show marginal effects associated with each level of covariate, interpreted as a percentage point difference in the probability of receiving palliative care or a difference in days enrolled in hospice. Each model also adjusted for state- and year-fixed effects (coefficients not shown).

^b^ Variables extracted from the Surveillance, Epidemiology, and End Results (SEER) registry.

^c^ Race and ethnicity information are abstracted from medical records using standardized fields by cancer registrars, but there may be misclassification of race and ethnicity by the reporting health care facilities and clinicians submitting such medical records. SEER provides separate variables for race and ethnicity, which we collapsed into the listed categories. The other category includes patients with non-Hispanic ethnicity and 1 or more of the following races: Alaska Native, Aleutian, American Indian, or Eskimo; Asian Indian or Pakistani; Chamorran; Chinese; Fiji Islander; Filipino; Guamanian; Hawaiian; Hmong; Japanese; Kampuchean; Korean; Laotian; Melanesian; Micronesian; New Guinean; Polynesian; Samoan; Tahitian; Thai; Tongan; Vietnamese; other Asian or Pacific Islanders; or other or unknown race.

^d^ Variables extracted from the linked Medicare enrollment or claims files.

^e^ We calculated the comorbidity index using all claims in the past 6 months of diagnosis; a higher index corresponds to higher comorbidities burden.

^f^ Yost Index of SES quintiles are characterized at the US Census tract level; the lowest quintile represents tracts with the lowest SES.

^g^ Rurality is characterized at the county level based on the share of the county population living in rural areas (>50%) from the 2010 US Census.

### Potentially Aggressive EOL Care in Last 30 Days of Life

**Figure 2.**
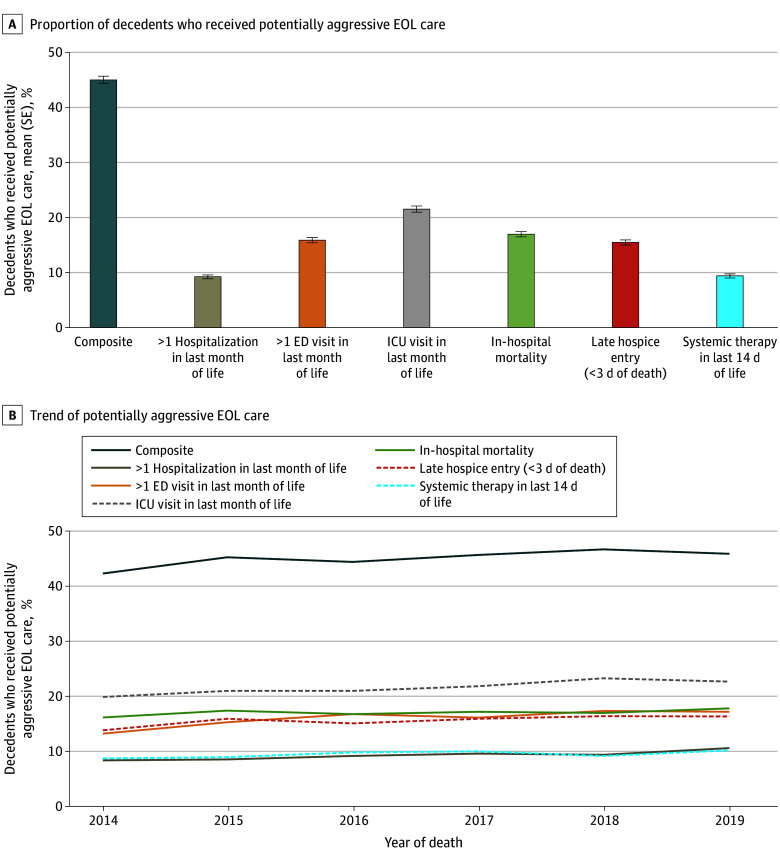
Prevalence and Yearly Trend of Claims-Based Indicators of Potentially Aggressive End-of-Life (EOL) Care Aggressive EOL care is defined as having multiple hospitalizations or emergency department (ED) visits or any intensive care unit visit in the last 30 days of life, dying in the hospital, entering hospice within 3 days of death (only among those who enrolled in hospice), and receipt of systemic therapy in the last 14 days of life. We limited the sample to those with 6 months of continuous Part D coverage because one of the measures is receipt of systemic therapy. The composite outcome equals 1 if a patient experienced any indicators of potentially aggressive EOL care. ICU indicates intensive care unit. Error bars indicate SEs.

Overall, 45.0% of the sample experienced any indicator of potentially aggressive EOL care (Figure 2), which was driven by intensive care unit visits. In general, the prevalence of potentially aggressive care was stable, without a clear change from 2014 to 2019. The prevalence was the highest among patients with lung cancer (47.7% for NSCL cancer and 47.1% for SCL cancer), followed by patients with breast (41.4%), pancreatic (41.3%), and prostate (37.6%) cancer (eFigure 4 in Supplement 1). In the adjusted model, patients with prostate and pancreatic cancer, respectively, had a 4.68–percentage point (95% CI, −7.17 to −2.19 percentage points; *P* < .001) and an 8.29–percentage point (95% CI, −10.06 to −6.52 percentage points; *P* < .001) lower probability of receiving potentially aggressive EOL care compared with patients with NSCL cancer (Table 3). Moreover, patients who lived 6 months or longer after diagnosis or were female, older, or unmarried were significantly less likely to receive potentially aggressive care. In contrast, non-Hispanic Black patients and those with greater comorbidity were more likely to receive potentially aggressive care. We found similar patterns when examining days in hospice in the last 30 days of life (Table 2). For example, patients with NSCL cancer had 1.84 (95% CI, 0.25-3.43; *P* = .02) and 2.30 (95% CI, 1.17-3.43; *P* < .001) fewer mean days in hospice compared with patients with prostate or pancreatic cancer, respectively.

**Table 3.** Patient Characteristics Associated With Receiving Potentially Aggressive End-of-Life Care

Characteristic	Adjusted marginal effect (95% CI)	*P* value
Cancer type		
NSCLC	1.0 [Reference]	NA
Breast	2.29 (−0.50 to 5.05)	.11
Prostate	−4.68 (−7.17 to −2.19)	<.001
Pancreas	−8.29 (−10.06 to −6.52)	<.001
SCLC	0.29 (−1.62 to 2.19)	.77
Time from diagnosis to death, mo		
<6	1.0 [Reference]	NA
6-18	−12.90 (−14.41 to −11.39)	<.001
>18	−15.18 (−17.00 to −13.38)	<.001
Sex^a^		
Male	1.0 [Reference]	NA
Female	−4.87 (−6.28 to −3.45)	<.001
Age at death, y		
66-70	1.0 [Reference]	NA
70-74	−3.33 (−5.11 to −1.54)	<.001
75-80	−5.02 (−6.89 to −3.14)	<.001
80-85	−13.96 (−15.79 to −12.13)	<.001
Race and ethnicity^a^^,^^b^		
Hispanic	2.55 (−0.39 to 5.49)	.09
Non-Hispanic Black	4.95 (2.55 to 7.35)	<.001
Non-Hispanic White	1.0 [Reference]	NA
Other or unknown	6.27 (3.47 to 9.07)	<.001
Marital status at diagnosis^a^		
Married	1.0 [Reference]	NA
Unmarried	−4.84 (−6.23 to −3.45)	<.001
Unknown	−4.52 (−7.73 to −1.31)	.006
Dually enrolled in Medicaid^c^		
No	1.0 [Reference]	NA
Yes	−1.63 (−3.25 to −0.003)	.050
NCI Comorbidity Index^c^^,^^d^		
0	1.0 [Reference]	NA
0-1	1.98 (0.54 to 3.42)	.007
1-2	3.18 (1.27 to 5.09)	.001
>3	5.72 (2.05 to 9.40)	.002
Yost Index of SES quintiles^a^^,^^e^		
1 (Low SES)	1.0 [Reference]	NA
2	2.26 (0.10 to 4.41)	.04
3	1.88 (−0.35 to 4.10)	.10
4	−0.97 (−3.27 to 1.33)	.41
5 (High SES)	−0.09 (−2.43 to 2.25)	.94
Unknown	0.37 (−4.23 to 4.97)	.87
Rurality^a^^,^^f^		
Metropolitan	1.0 [Reference]	NA
Urban	1.86 (−0.30 to 4.04)	.09
Rural	2.36 (−0.55 to 5.27)	.11

Abbreviations: NA, not applicable; NCI, National Cancer Institute; NSCLC, non–small cell lung cancer; SCLC, small cell lung cancer; SES, socioeconomic status.

^a^ Variables extracted from the Surveillance, Epidemiology, and End Results (SEER) registry.

^b^ Race and ethnicity information are abstracted from medical records using standardized fields by cancer registrars, but there may be misclassification of race and ethnicity by the reporting health care facilities and clinicians submitting such medical records. SEER provides separate variables for race and ethnicity, which we collapsed into the listed categories. The other category includes patients with non-Hispanic ethnicity and 1 or more of the following races: Alaska Native, Aleutian, American Indian, or Eskimo; Asian Indian or Pakistani; Chamorran; Chinese; Fiji Islander; Filipino; Guamanian; Hawaiian; Hmong; Japanese; Kampuchean; Korean; Laotian; Melanesian; Micronesian; New Guinean; Polynesian; Samoan; Tahitian; Thai; Tongan; Vietnamese; other Asian or Pacific Islanders; or other or unknown race.

^c^ Variables extracted from the linked Medicare enrollment or claims files.

^d^ We calculated the comorbidity index using all claims in the past 6 months of diagnosis; a higher index corresponds to higher comorbidities burden.

^e^ Yost Index of SES quintiles are characterized at the US Census tract level; the lowest quintile represents tracts with the lowest socioeconomic status.

^f^ Rurality is characterized at the county-level based on the share of the county population living in rural areas (>50%) from the 2010 US Census.

## Discussion

In this large retrospective cohort study of older FFS Medicare decedents originally diagnosed with advanced female breast, prostate, lung, or pancreatic cancer, we report EOL care patterns during the period when considerable efforts have focused on improving the quality of EOL care.^2,4,5,6^ Despite these efforts, we observed consistent evidence of potentially aggressive care, including a high rate of acute care and systemic therapies near death, low uptake of PC and ACP, and late hospice enrollment, largely replicating findings from earlier investigations in prior decades.^2,5,6,14,35,36^

What could explain the enduring stability in EOL care patterns in oncology? The answer requires us to acknowledge the fundamental challenge faced by care stakeholders (ie, patients, their families, and clinicians) in making care decisions at EOL: accurately assessing the harms and benefits of treatment amidst prognostic uncertainty. Our results suggest that care continues to favor overtreatment, even though the awareness of aggressive cancer treatment has increased over time. One reason for overtreatment may be the proliferation of novel anticancer therapies (especially targeted and immunotherapies),^37,38,39,40^ promising marked improvements in survival and yet complicating an accurate prognostic assessment. These therapies may inculcate a belief that a metastatic cancer may be curable, leading to potentially aggressive care despite patient preferences for initiating EOL care.^40^ Consistent with this hypothesis, we observed the highest rate of potentially aggressive care among patients with metastatic lung cancer, the disease area experiencing a disproportionate increase in the number of newly available innovative treatments during the study period.^37^ On the other hand, patients with a longer survival duration (such as those diagnosed with prostate cancer) were less likely to receive potentially aggressive care. It is plausible that longer survival time after diagnosis may provide more ample opportunities to initiate EOL care discussions and facilitate more realistic expectations about prognosis, which are factors associated with less intensive care.^41,42,43^

Overall, our study underscores the importance of clear communication about disease prognosis, EOL care options, and patient preferences.^35,44,45,46^ Prior research suggests that clinicians’ beliefs and practice styles were the largest drivers of the intensity of EOL care, meaning that clinicians may play an outsized role in conversations about EOL care.^35^ These conversations should recognize that most Medicare beneficiaries with advanced cancer prefer to die in their homes in the community and receive palliative, rather than life-extending, care in hospital settings.^47,48,49^ Given considerable health services use at EOL, there are many avenues to initiate EOL care planning at points of contact with the health care system. For instance, exposure to EOL care planning during an ED visit can significantly reduce inpatient use and the likelihood of in-hospital deaths among critically ill Medicare patients.^50^

On the other hand, low rates of supportive care, including PC and HC, are concerning because they suggest considerable unmet quality-of-life needs. Recognizing the well-established benefits of PC,^51,52,53,54^ ASCO issued a provisional clinical opinion recommending concurrent PC with usual oncology care for all patients with advanced cancer in 2012, which was then adopted as a full practice guideline in 2016 and updated in 2024.^55^ Contrary to this recommendation, only one-quarter of patients in our sample received any PC in the last 6 months of life, and those who received PC did so mainly in the month of death, similar to the findings of prior studies.^56,57,58^ It is possible that the ASCO recommendation is yet to be adopted widely in clinical practice, as indicated by the gradually increasing uptake of PC over time. However, gaps in PC use may also reflect stigma about palliative care (ie, a belief that PC is synonymous to “giving up” on a cure^59^) or access barriers, such as inadequate supply of PC clinicians.^2,60,61,62^ Further clarifying the purpose and benefits of PC, along with concrete actions to ensure adequate availability of PC clinicians, may be essential to improve use of PC, especially among patients who disproportionately experience access problems (eg, those living in rural areas) and patients with more severe symptom burden and comorbidities.

Furthermore, we documented a familiar pattern of hospice underuse and late enrollment.^43,63,64,65,66^ For dying patients and their caregivers, hospice is often considered the gold standard of EOL care that can holistically manage care needs.^65,67^ The fact that a considerable portion did not use HC at all or entered hospice within 3 days of death suggests the potential benefits of HC were not realized for many patients. Although not a direct focus of our study, there are relevant system-level factors that are worth noting in contextualizing this pattern of HC. First, the eligibility criteria for the Medicare Hospice Benefit strictly require that patients waive all treatment with curative intent, imposing an arbitrary clinical distinction between curative and supportive care that can discourage hospice entry.^68^ Second, the low–per diem coverage within the benefit may lead hospice programs to underenroll patients with cancer, who often require costly services relative to patients with other serious illnesses.^67,69,70^ The increase in for-profit hospice programs exacerbates these concerns about access because they face even greater financial incentives to reduce costs.^71,72,73^ These factors warrant considerations for a concurrent coverage of curative and supportive care within Medicare^74,75,76,77^ and an increased level of reimbursement to support enrollment of patients with advanced cancer.^78^

One unique service type that we included in our evaluations of EOL care (in contrast to prior studies^2,4,5,6^) was the receipt of ACP, which recently became a billable service to Medicare.^13,79,80^ In our sample, only one-tenth had any ACP visits, and such visits were concentrated in the month of death, meaning that care planning often occurred too late in a patient’s care journey, if it occurred at all. Given that patients and their families often have a desire for ACP,^48,81,82,83^ our finding may suggest clinician-related barriers to ACP. For instance, lack of awareness of the new billing code, documentation burden, and low reimbursement rates ($80-86 per 30 minutes of ACP) have contributed to reduced willingness and abilities to administer ACP.^79^ Further monitoring of these barriers may help guide future reforms to ACP reimbursement.

### Limitations

We acknowledge several limitations to this study. First, our data lacked several important determinants of EOL care, such as patient preferences for treatment, symptom burden, functional status, and detailed characteristics of care settings (eg, receipt of care at accredited cancer centers), which should be incorporated in future studies. Second, the claims-based measures of potentially aggressive care may not account for the appropriateness of care in specific clinical contexts. Furthermore, we assessed these measures retrospectively from the point of death (which is unknown in advance), raising questions as to whether our results should be interpreted as a marker of true “poor” care quality.^84,85^ Nonetheless, our sample consisted of patients diagnosed with advanced cancer for whom one can reasonably infer limited life expectancy and high supportive care needs, even at diagnosis. In this population, we examined use in a short window before death (when patients are likely to exhibit significant clinical decline), which can provide useful insights into care decision-making. Third, the sample included only 5 cancer types, but these cancers contribute to more than 50% of all cancer deaths^86^ and vary in key clinical characteristics, such as length of survival,^87^ allowing us to examine the heterogeneity of care patterns. Fourth, it is possible that underlying cause of death may have been miscoded, although prior validation studies have found a high congruence between the coded cause of death with initial cancer diagnoses in cancer registries.^23,24,25^ Fifth, we measured comorbidities before diagnosis to ensure that we do not misclassify treatment-related complications and adverse effects as comorbidities.^88,89,90,91,92^ This approach may miss other chronic conditions developed after diagnosis that may be correlated with EOL care. Sixth, we excluded Medicare Advantage beneficiaries whose claims data were unavailable. Understanding care patterns in Medicare Advantage beneficiaries is an important avenue for future research because enrollment in Medicare Advantage is projected to continue to rapidly increase.^93^

## Conclusions

In a contemporary cohort of older Medicare decedents originally diagnosed with advanced breast, prostate, pancreatic, or lung cancer, we found that many patients continue to receive potentially aggressive interventions at EOL at the expense of supportive care services. To make meaningful improvements in the quality of EOL care, a multifaceted approach that addresses patient, physician, and system-level factors associated with persistent patterns of potentially aggressive care will be required.

## Notes

Supplement 1. **eTable 1.** Constructing study cohort of older, fee-for-service Medicare beneficiaries who died due to advanced breast, lung, prostate, or pancreatic cancers from 2014 to 2019 **eTable 2.** Histology codes to identify primary histology type for each cancer **eTable 3.** Algorithm to construct end-of-life healthcare utilization outcomes **eTable 4.** Characteristics of older, fee-for-service Medicare beneficiaries diagnosed with advanced cancer 2008 to 2019 and dying from cancer from 2014 to 2019 in SEER-Medicare, by year of death **eFigure 1.** Study sample by year of diagnosis **eFigure 2.** Trajectories of hospitalization care in the last 6 months among older fee-for-service Medicare beneficiaries diagnosed with advanced cancer 2008 to 2019 and dying from cancer 2014 to 2019 in SEER-Medicare, by cancer type **eFigure 3.** Yearly trend in receipt of palliative care among older fee-for-service Medicare beneficiaries diagnosed with advanced cancer 2008 to 2019 and dying from cancer from 2014 to 2019 in SEER-Medicare **eFigure 4.** Prevalence of claims-based indicators of potentially aggressive EOL care among older fee-for-service Medicare beneficiaries diagnosed with advanced cancer 2008 to 2019 and dying from cancer 2014 to 2019 in SEER-Medicare, by cancer type

Supplement 2.
Data Sharing Statement
