# Supplementary material for: Contemporary Patterns of End-of-Life Care Among Medicare Beneficiaries With Advanced Cancer
Source: JAMA Health Forum. 2025 Feb 21;6(2):e245436. doi: 10.1001/jamahealthforum.2024.5436 (PMC11846012; doi:10.1001/jamahealthforum.2024.5436)
Supplement: Supplement 1. — eTable 1. Constructing study cohort of older, fee-for-service Medicare beneficiaries who died due to advanced breast, lung, prostate, or pancreatic cancers from 2014 to 2019 eTable 2. Histology codes to identify primary histology type for each cancer eTable 3. Algorithm to construct end-of-life healthcare utilization outcomes eTable 4. Characteristics of older, fee-for-service Medicare beneficiaries diagnosed with advanced cancer 2008 to 2019 and dying from cancer from 2014 to 2019 in SEER-Medicare, by year of death eFigure 1. Study sample by year of diagnosis eFigure 2. Trajectories of hospitalization care in the last 6 months among older fee-for-service Medicare beneficiaries diagnosed with advanced cancer 2008 to 2019 and dying from cancer 2014 to 2019 in SEER-Medicare, by cancer type eFigure 3. Yearly trend in receipt of palliative care among older fee-for-service Medicare beneficiaries diagnosed with advanced cancer 2008 to 2019 and dying from cancer from 2014 to 2019 in SEER-Medicare eFigure 4. Prevalence of claims-based indicators of potentially aggressive EOL care among older fee-for-service Medicare beneficiaries diagnosed with advanced cancer 2008 to 2019 and dying from cancer 2014 to 2019 in SEER-Medicare, by cancer type [file jamahealthforum-e245436-s001.pdf]

Supplemental Online Content

Kwon Y, Hu X, Shi KS, et al. Contemporary patterns of end-of-life care among Medicare beneficiaries with advanced cancer. *JAMA Health Forum*. Published online February 21, 2025. doi:10.1001/jamahealthforum.2024.5436

eTable 1. Constructing study cohort of older, fee-for-service Medicare beneficiaries who died due to advanced breast, lung, prostate, or pancreatic cancers from 2014 to 2019

eTable 2. Histology codes to identify primary histology type for each cancer

eTable 3. Algorithm to construct end-of-life healthcare utilization outcomes

eTable 4. Characteristics of older, fee-for-service Medicare beneficiaries diagnosed with advanced cancer 2008 to 2019 and dying from cancer from 2014 to 2019 in SEER-Medicare, by year of death

eFigure 1. Study sample by year of diagnosis

eFigure 2. Trajectories of hospitalization care in the last 6 months among older fee-for-service Medicare beneficiaries diagnosed with advanced cancer 2008 to 2019 and dying from cancer 2014 to 2019 in SEER-Medicare, by cancer type

eFigure 3. Yearly trend in receipt of palliative care among older fee-for-service Medicare beneficiaries diagnosed with advanced cancer 2008 to 2019 and dying from cancer from 2014 to 2019 in SEER-Medicare

eFigure 4. Prevalence of claims-based indicators of potentially aggressive EOL care among older fee-for-service Medicare beneficiaries diagnosed with advanced cancer 2008 to 2019 and dying from cancer 2014 to 2019 in SEER-Medicare, by cancer type

This supplemental material has been provided by the authors to give readers additional information about their work.

© 2025 Kwon Y et al. *JAMA Health Forum*.

eTable 1 Constructing study cohort of older, fee-for-service Medicare beneficiaries who died due to advanced breast, lung, prostate, or pancreatic cancers from 2014 to 2019

	N, remaining after applying each criterion				
**Inclusion criteria**	**Overall**	**Breast**	**Lung**	**Prostate**	**Pancreas**
All cases from SEER registry	3,490,928	1,155,766	1,057,024	1,057,023	221,115
Only one lifetime cancer diagnosis[a]	2,024,142	633,354	558,967	697,466	134,355
Matching death information in SEER & Medicare[b]	468,259	90,618	220,257	95,233	62,151
Year of diagnosis from 2008 to 2019[c]	447,640	83,176	218,011	84,471	61,982
Age at diagnosis 66 to 85	368,091	64,581	181,107	69,649	52,754
Death from 2014-2019	222,014	38,594	108,728	42,717	31,975
Survive at least 30 days post-diagnosis[d]	197,567	36,624	94,381	39,479	27,083
Stage 4 (AJCC) or distant-stage cancers only[e]	81,002	5,033	52,348	9,723	13,898
6 mos. continuous enrollment in FFS Medicare A & B	45,871	2,997	29,827	5,381	7,666
Primary histology groups[f]	40,455	5,407	24,909	4,285	5,854
Died due to cancer-specific causes[g]	33,744	2,086	22,824	3,239	5,595
Final sample	33,744	2,086	22,824	3,239	5,595

Abbreviations: SEER, Surveillance, Epidemiology, and End Results Program; AJCC, American Joint Committee on Cancer; Mos, months; FFS, fee-for-service

[a] Cases with sequence number (SEQUENCE_NUMBER)= "00", indicating no multiple cancer diagnoses in lifetime.

[b] We only included cases who died and had the same date of death in both SEER registry and the linked Medicare beneficiary files ("Date of Death Flag = 1 Dead, both files agree").

[c] We excluded those diagnosed in 2007 as most of them would not have sufficient pre-diagnosis data which we use to examine continuous enrollment criteria and characterize baseline covariates (our SEER-Medicare data spanned years 2007 to 2020). We used the SEER_DateofDeath_Year variable to identify year of death.

[d] We used death date in the linked Medicare beneficiary files (BENE_DEATH_DT) because it provides the exact date of death, rather than death month provided in SEER.

[e] AJCC derived stage variable (DerivedAJCCStageGroup7thed_2010 or DerivedAJCCStageGroup6thed20042) was available for most cases who were diagnosed in 2015 or earlier. For cases with missing AJCC staging variable (this includes cases who were diagnosed after 2015), we included cases whose SEER summary stage (SEERCOMBINEDSUMMARYSTAGE2000200) indicated distant stage cancer at diagnosis. In cases with both staging variables available, more than 90% of cases with AJCC Stage IV cancer were categorized as having distant stage as their summary stage, indicating a high agreement between these two staging variables.

[f] To ensure representativeness, we only included patients with primary histology types (HISTOLOGIC_TYPE_ICD_O_3) for breast, prostate, pancreatic, non-small cell lung, and small cell lung cancers using ICD-O-3 codes (3rd edition International Classification of Disease for Oncology). See Table S2 for a full list of the histology codes we used.

[g] We used the cause-specific death variable in SEER (i.e., where SEERcausespecificdeathclassific = 1 and SEERothercauseofdeathclassifica = 0), which attributes causes of death from death certificates based on an algorithm that considers tumor sequence, site of original cancer diagnosis, and comorbidities.[1]

© 2025 Kwon Y et al. *JAMA Health Forum*.

eTable 2 Histology codes to identify primary histology type for each cancer

Cancer type	Included histology types	ICD-O-3 histology codes
Breast	Adenocarcinoma NOS, duct carcinoma, lobular and other ductal carcinoma, adenocarcinoma with metaplasia	8140, 8141, 8143, 8147, 8500, 8501, 8502, 8503, 8504, 8507, 8508, 8509, 8520, 8521, 8522, 8523, 8524, 8525, 8570, 8571, 8572, 8573, 8574, 8575
Prostate	Adenocarcinoma NOS	8140
Pancreas	Adenocarcinoma NOS, scirrhous adenocarcinoma, superficial spreading adenocarcinoma, tubular adenocarcinoma, invasive carcinoma of no special type, comedocarcinoma, NOS, intraductal papillary adenocarcinoma with invasion, intracystic carcinoma, NOS, cystic hypersecretory carcinoma	8140, 8141, 8143, 8211, 8500, 8501, 8503, 8504, 8508
NSCLC	Non-small cell carcinoma NOS, papillary carcinoma NOS, squamous cell carcinoma, NOS, adenocarcinoma NOS, bronchiolo-alveolar adenoca, papillary adenocarcinoma NOS, clear cell adenocarcinoma NOS, mucoepidermoid carcinoma, mucinous adenocarcinoma, adenosquamous carcinoma, adenocarcinoma with metaplasia	8046, 8050, 8051, 8052, 8070, 8071, 8072, 8073, 8074, 8075, 8076, 8078, 8140, 8141, 8143, 8147, 8250, 8251, 8252, 8253, 8254, 8255, 8260, 8310, 8430, 8480, 8481, 8560, 8570, 8571, 8572, 8573, 8574
SCLC	Small cell carcinoma, NOS, oat cell carcinoma, small cell carcinoma (fusiform cell), small cell carcinoma (intermediate cell), combined small cell carcinoma	8041, 8042, 8043, 8044, 8045

Abbreviations: ICD-O, International Classification of Diseases for Oncology, NSCLC, non-small cell lung cancer; SCLC, small-cell lung cancer

© 2025 Kwon Y et al. *JAMA Health Forum*.

eTable 3 Algorithm to construct end-of-life healthcare utilization outcomes

Utilization type	Medicare files used	Identification algorithm
**Hospitalizations**	• MedPAR[a]	We counted the number of hospitalization episodes (each episode consists of consecutive hospital visit days with a gap of less than 2 days). We only used claims in MedPAR with NCH claims type code 60 or 61 (Medicare inpatient claims). We excluded hospital admission occurring after hospice enrollment.
**ER visits**	• MedPAR,<br>• Outpatient	We used the ResDAC algorithm, flagging ER visits in outpatient files using the Revenue Center Codes 0450-0459 and 0981 and inpatient claims with emergency room charge amount > $0 in MedPAR.[2] We excluded any ER visits occurring after hospice enrollment.
**Systemic therapies**	• MedPAR<br>• Outpatient<br>• Professional carrier<br>• Part D event<br>• DME	In inpatient, outpatient, and professional carrier claims, we flagged infused chemotherapy using diagnosis, procedure, and revenue center codes (see Table S4 for a full list). To identify oral chemotherapy, we used Medicare Part D event and DME files (DME files may contain claim for oral chemotherapy drugs that are equivalent to Part B infused chemotherapy drugs[3]) and identify chemotherapy using NDC codes appearing in CanMED.[3] We exclude patients without continuous Part D enrollment during 6 months before death from the denominator because we examine receipt of Part B and D chemotherapy.
**Hospice**	• Hospice	We count hospice enrollment once, flagging the earliest claim for hospice in the hospice file. We assume that patients remain in hospice upon entry, since disenrollment from hospice is likely rare among patients with advanced cancer.
**Palliative care**	• MedPAR<br>• Outpatient<br>• Professional carrier	We identified palliative care provided outside of hospice settings in inpatient, outpatient, and carrier claims using ICD diagnosis code indicating encounter for palliative care (ICD-9: V66.7; ICD-10: Z51.5) and provider specialty code number for hospice and palliative care providers (17). When identifying palliative care claims using the provider specialty code, we exclude claims that occur within 7 days of hospice enrollment period.
**Advanced care planning**	• Outpatient<br>• Professional carrier	We flagged any outpatient or carrier claims with the CPT codes for advanced care planning (99497 or 99498). We only evaluate advanced care planning use among patients dying in or after 2016 when the CMS began reimbursing advance care planning services for fee-for-service Medicare beneficiaries.[4]

Abbreviations: MedPAR, Medicare Provider Analysis and Review; NCH, National Claims History; ER, emergency room; ResDAC, Research Data Assistance Center; DME, durable medical equipment; NDC, National Drug Code; CanMED, Cancer Medications Enquiry Database; ICD, International Classification of Diseases; CPT, Current Procedural Terminology; CMS, Centers for Medicare and Medicaid

© 2025 Kwon Y et al. *JAMA Health Forum*.

eTable 4 Characteristics of older, fee-for-service Medicare beneficiaries diagnosed with advanced cancer 2008 to 2019 and dying from cancer from 2014 to 2019 in SEER-Medicare, by year of death

Characteristic[a]	2014 N = 5,767	2015 N = 5,708	2016 N = 5,818	2017 N = 5,757	2018 N = 5,562	2019 N = 5,132
**Sex, %**						
Male	51.1	53.0	52.4	52.0	52.2	52.0
Female	48.9	47.0	47.6	48.0	47.8	48.0
**Age at diagnosis, %**						
66-69	21.5	22.5	22.0	23.0	22.0	20.1
70-74	26.7	26.9	27.0	26.6	26.7	27.0
75-79	22.6	22.1	22.1	21.7	22.5	23.1
80-85	29.2	28.5	28.9	28.7	28.8	29.8
**Race and ethnicity, %[b]**						
Hispanic	5.2	5.0	4.8	5.0	5.2	4.8
Non-Hispanic White	80.3	80.8	80.7	80.7	79.9	80.7
Non-Hispanic Black	9.6	8.9	9.1	8.8	8.8	8.2
Other/unknown	4.8	5.3	5.4	5.5	6.1	6.2
**Marital status, %[c]**						
Single	10.6	9.4	10.4	10.6	11.6	11.8
Married	49.1	50.6	50.6	51.8	50.6	50.1
Separated, divorced, widowed	36.0	35.8	34.9	33.3	33.8	33.5
Unknown	4.3	4.2	4.1	4.3	4.0	4.7
**Dually enrolled in Medicaid, %[d]**						
Any dual enrollment	19.8	19.6	19.3	19.5	20.1	19.5
No dual enrollment	80.2	80.4	80.7	80.5	79.9	80.5
**Part D enrollment, %**						
Continuously enrolled	68.4	68.8	69.0	70.1	69.7	70.7
No continuous enrollment	31.6	31.2	31.0	29.9	30.3	29.3
**Duration of survival, %[e]**						
<6 months	55.9	56.4	55.7	54.2	52.7	52.5
6 to 12 months	16.1	15.3	15.5	15.6	15.0	15.1
12 to 18 months	10.6	10.5	10.0	10.3	9.9	10.7
18 to 24 months	5.7	5.7	5.9	5.8	6.8	6.8
24 to 36 months	6.1	5.8	5.8	6.2	7.1	6.8
36 to 48 months	2.7	2.8	3.0	3.2	3.4	3.2
48 to 60 months	1.7	1.4	1.7	1.9	2.1	2.0
60+ months	1.2	2.0	2.3	2.8	3.0	2.9
**NCI comorbidities index, %[f]**						
0	37.0	37.6	36.4	37.0	36.8	36.1
0-1	46.2	44.2	44.8	44.8	42.7	43.4
1-2	14.6	15.6	16.0	15.4	16.9	17.2
2+	2.2	2.5	2.8	2.9	3.6	3.3
**Yost Index quintile, %[g]**						
Quintile 1 (low SES)	18.3	18.7	18.2	18.4	17.4	16.3
Quintile 2	19.0	17.6	18.0	16.9	17.7	17.8
Quintile 3	18.4	18.4	18.7	18.8	18.9	19.4
Quintile 4	19.9	20.3	19.9	20.4	19.7	21.1
Quintile 5 (high SES)	22.4	22.9	23.3	23.5	24.4	23.3
Missing	2.0	2.2	1.9	2.0	2.0	2.1
**Rurality, %[h]**						
Metropolitan	82.6	81.1	81.7	81.3	81.4	81.7
Urban	11.8	12.0	10.9	12.2	12.0	12.0
Rural	5.6	6.9	7.4	6.5	6.6	6.4
**SEER cancer registry, %**						

© 2025 Kwon Y et al. *JAMA Health Forum*.

California	29.6	29.3	28.9	29.8	29.6	30.2
Connecticut	5.7	5.2	5.7	6.5	4.9	4.9
Georgia	12.5	12.0	12.3	11.7	12.7	12.4
Iowa	6.5	7.5	6.8	7.2	7.1	7.2
Kentucky	8.9	9.3	9.2	9.0	9.1	9.3
Louisiana	6.9	6.3	6.2	6.3	6.2	6.7
Detroit	7.7	6.2	6.6	6.4	6.2	5.9
New Jersey	13.4	14.3	14.7	13.0	14.0	12.7
New Mexico	2.2	2.2	1.8	2.0	1.7	1.8
Utah	1.2	1.7	1.7	1.8	1.9	2.0
Seattle	5.3	6.0	6.1	6.2	6.6	6.9

Abbreviations: NSCLC, non-small cell lung cancer; SCLC, small cell lung cancer; NCI, National Cancer Institute; SEER, Surveillance, Epidemiology, and End Results

[a] We report characteristics of fee-for-service Medicare beneficiaries older than age 65 who died from Stage IV or distant stage breast, prostate, pancreas, non-small cell lung or small cell lung cancer from 2014 to 2019 in SEER-linked Medicare files by year of death.

[b] Race and ethnicity information are abstracted from medical records using standardized fields by cancer registrars, but there may be misclassification of race and ethnicity by the facilities and providers submitting such medical records. SEER provides separate variables for race and ethnicity, which we collapsed into the listed categories. "Other" category includes cases with non-Hispanic ethnicity and one or more of the following race: American Indian/Aleutian/Alaskan Native or Eskimo, Chinese, Japanese, Filipino, Hawaiian, Korean, Vietnamese, Laotian, Hmong, Kampuchean, Thai, Asian Indian or Pakistani, Micronesian, Chamorran, Guamanian, Polynesian, Tahitian, Samoan, Tongan, Melanesian, Fiji Islander, New Guinean, Other Asian or Pacific Islanders, "Other", or unknown race.

[c] Marital status at the time of diagnosis that are self-reported on medical charts. SEER's definition of common law marriage includes a couple living together and declaring themselves as married, not requiring any formal ceremony or a marriage license.[5]

[d] We identified dual enrollment using the Medicaid buy-in codes from the linked Medicare master beneficiary files, including both partial or full Medicaid coverage.

[e] We calculated months from diagnosis to death using the death date in the linked Medicare files that provides the exact date of death, as opposed to death month (as is the case in SEER); all cases in the sample had agreeing death date information in both SEER and the linked Medicare files.

[f] The NCI comorbidity index is a modified version of the Charlson Comorbidity Index that excludes diagnoses of solid tumors, leukemias, and lymphoma. We calculated the index using Medicare Part A and B claims occurring within 6 months prior to diagnosis.

[g] We characterized the Yost Index quintile variable provided by SEER, which is calculated at the Census tract level using the 2013-2014 American Community Survey; the first (fifth) quintile is the Census tract group with the lowest (highest) socioeconomic status.

[h] We categorized the Census tract-level rurality based on the identification of urban and rural areas from the 2010 Census; "Urban" denotes Census tract with more than 50% share of the population living in an urban area.

© 2025 Kwon Y et al. *JAMA Health Forum*.

eFigure 1 Study sample by year of diagnosis

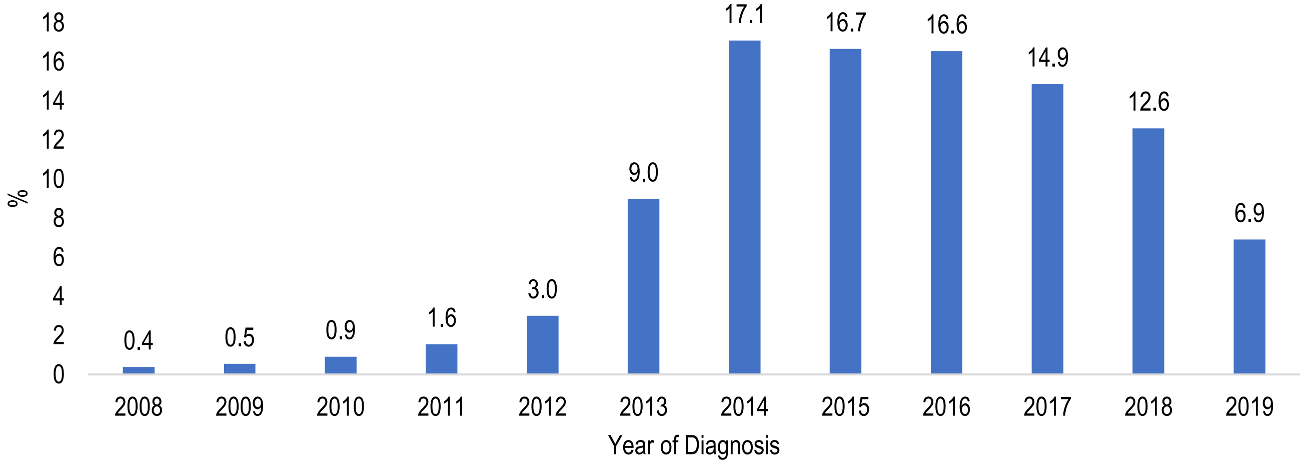

Note: Figure displays the share of Medicare decedents dying from advanced cancer in the sample by year of diagnosis.

© 2025 Kwon Y et al. *JAMA Health Forum*.

eFigure 2 Trajectories of hospitalization care in the last 6 months among older fee-for-service Medicare beneficiaries diagnosed with advanced cancer 2008 to 2019 and dying from cancer 2014 to 2019 in SEER-Medicare, by cancer type

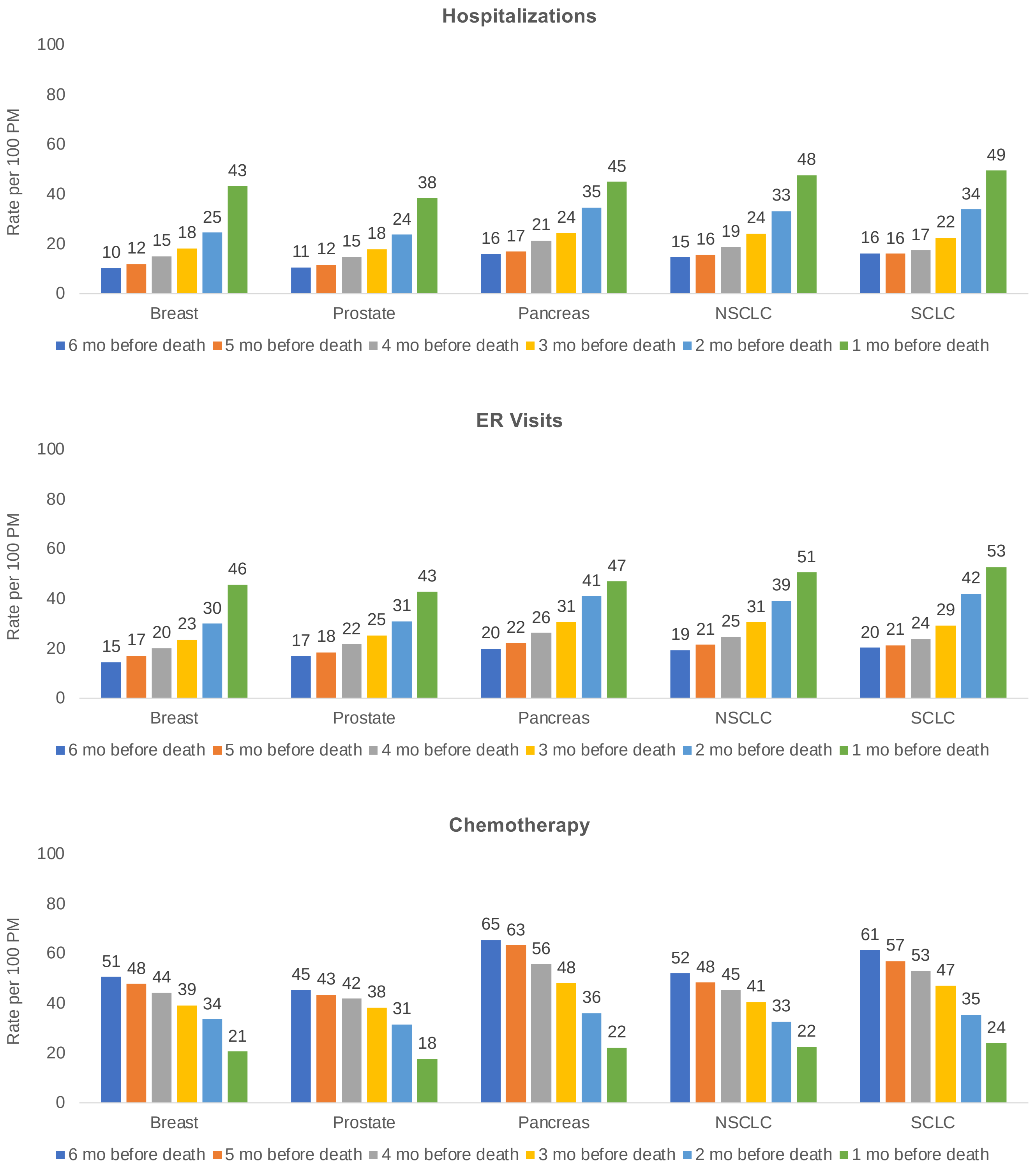

© 2025 Kwon Y et al. *JAMA Health Forum*.

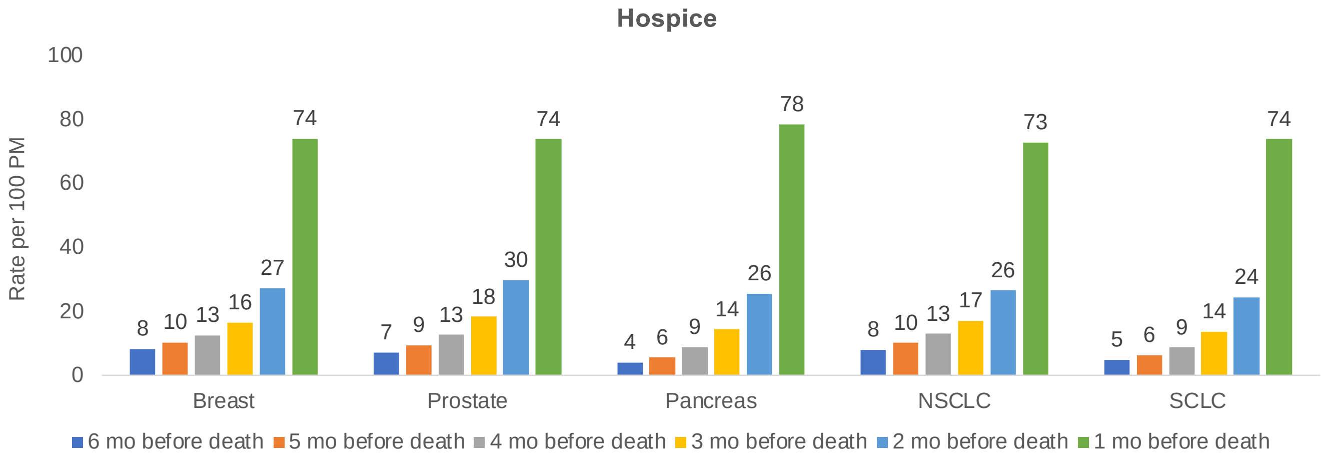

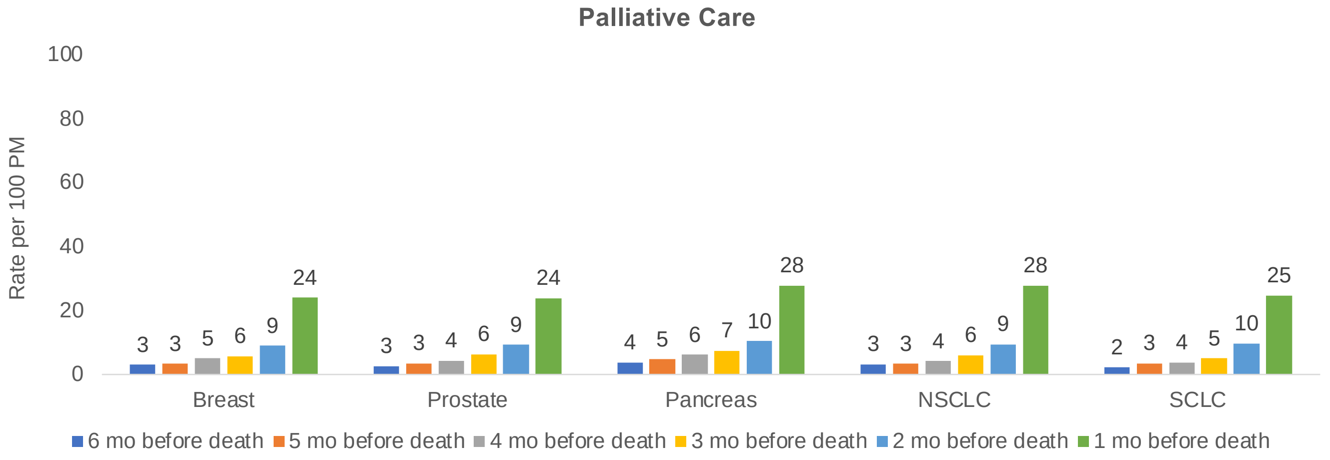

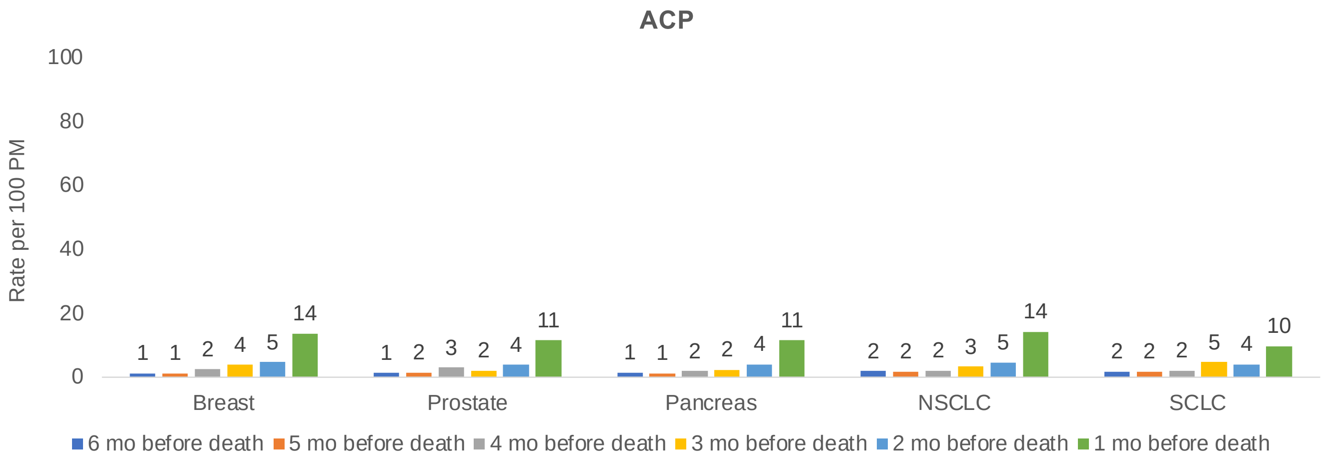

Abbreviations: PM, person-months; ER, emergency room; Mo, months; ACP, advanced care planning
Note: This figure reports mean monthly rates of healthcare utilization from 6-months prior to death to the month of death for each service type, separately for each cancer type. The rate is calculated as the number of patients utilizing healthcare service divided by the person-days when patients were alive and diagnosed with cancer, converted to a person-months denominator (this accounts for the fact that not all patients survived at least 6 months). The denominator for chemotherapy excluded patients without continuous Part D enrollment in the last 6 months of life. Palliative care refers to supportive care provided outside of hospice. ACP utilization was only measured for the sample dying at or after 2016.

© 2025 Kwon Y et al. *JAMA Health Forum*.

eFigure 3 Yearly trend in receipt of palliative care among older fee-for-service Medicare beneficiaries diagnosed with advanced cancer 2008 to 2019 and dying from cancer from 2014 to 2019 in SEER-Medicare

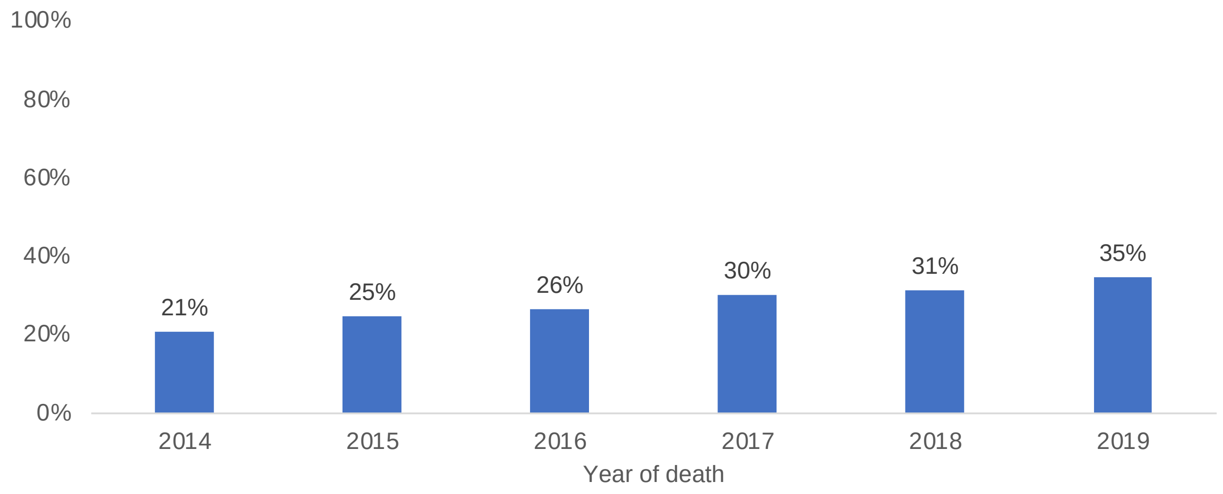

Note: Figure displays the share of Medicare decedents dying from advanced care receiving palliative care in each year. We only measured palliative care provided outside of hospice (i.e., outpatient palliative care).

© 2025 Kwon Y et al. *JAMA Health Forum*.

eFigure 4 Prevalence of claims-based indicators of potentially aggressive EOL care among older fee-for-service Medicare beneficiaries diagnosed with advanced cancer 2008 to 2019 and dying from cancer 2014 to 2019 in SEER-Medicare, by cancer type

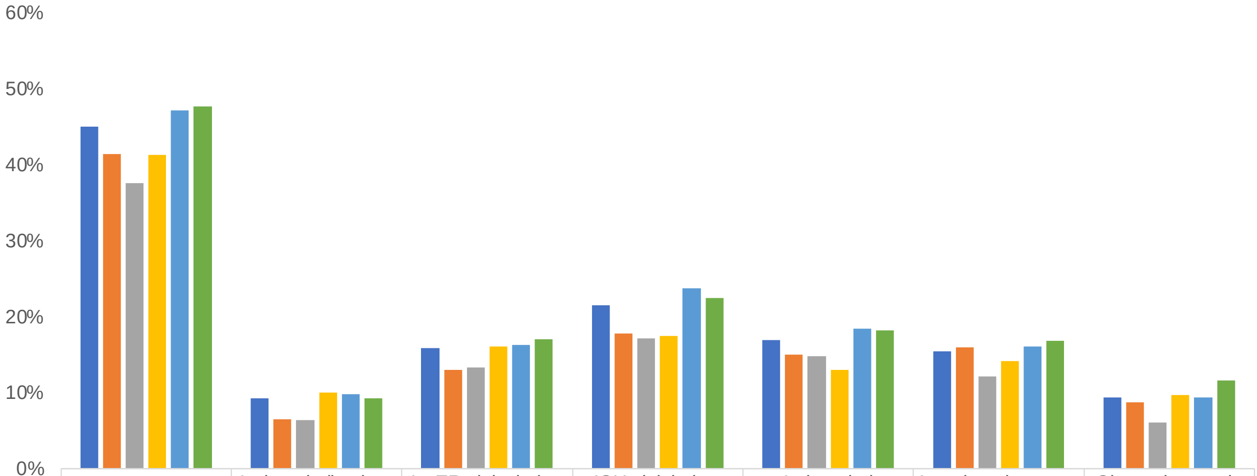

	Composite	1> hospitalizations in last month	1> ER visits in last month	ICU visit in last month	In-hospital mortality	Late hospice entry (<3 days of death)	Chemotherapy in last 14 days
Overall	45%	9%	16%	22%	17%	15%	9%
Breast	41%	7%	13%	18%	15%	16%	9%
Prostate	38%	6%	13%	17%	15%	12%	6%
Pancreas	41%	10%	16%	17%	13%	14%	10%
NSCLC	47%	10%	16%	24%	18%	16%	9%
SCLC	48%	9%	17%	22%	18%	17%	12%

Abbreviations: EOL, end-of-life; ER, emergency room; ICU, intensive care unit; NSCLC, non-small cell lung cancer; SCLC, small cell lung cancer

Note: Figures display the overall share of Medicare decedents dying from advanced care receiving potentially aggressive EOL care, defined as having multiple hospitalizations or ER visits, or any ICU visit in last 30 days, dying in hospital, entering hospice within 3 days of death (only among those who enrolled in hospice), and receipt of chemotherapy in last 14 days. We report the indicators separately by cancer type. We limited the sample to those with 6 months of continuous Part D coverage, as one of the measures is receipt of chemotherapy. The composite outcome equals 1 if a patient experienced any indicators of potentially aggressive EOL care.

© 2025 Kwon Y et al. *JAMA Health Forum*.

eReferences

1. Howlader N, Ries LA, Mariotto AB, Reichman ME, Ruhl J, Cronin KA. Improved estimates of cancer-specific survival rates from population-based data. *J Natl Cancer Inst*. Oct 20 2010;102(20):1584-98. doi:10.1093/jnci/djq366
2. ResDAC. How to Identify Hospital Claims for Emergency Room Visits in the Medicare Claims Data. Jul 30, 2015. Accessed Dec 21, 2023. https://resdac.org/articles/how-identify-hospital-claims-emergency-room-visits-medicare-claims-data
3. Lund JL, Stürmer T, Harlan LC, et al. Identifying specific chemotherapeutic agents in Medicare data: a validation study. *Med Care*. May 2013;51(5):e27-34. doi:10.1097/MLR.0b013e31823ab60f
4. Palmer MK, Jacobson M, Enguidanos S. Advance Care Planning For Medicare Beneficiaries Increased Substantially, But Prevalence Remained Low. *Health Aff (Millwood)*. Apr 2021;40(4):613-621. doi:10.1377/hlthaff.2020.01895
5. National Cancer Institute. SEER Program, Coding and Staging Manual 2023. Sep, 2022. Accessed May 25, 2024. https://seer.cancer.gov/manuals/2023/SPCSM_2023_MainDoc.pdf

© 2025 Kwon Y et al. *JAMA Health Forum*.
