# Supplementary material for: Contemporary Patterns of End-of-Life Care Among Medicare Beneficiaries With Advanced Cancer
Source: JAMA Health Forum. 2025 Feb 21;6(2):e245436. doi: 10.1001/jamahealthforum.2024.5436 (PMC11846012; doi:10.1001/jamahealthforum.2024.5436)
Supplement: Supplement 2. — Data Sharing Statement [file jamahealthforum-e245436-s002.pdf]

Data Sharing Statement

Kwon. Contemporary Patterns of End-of-Life Care Among Medicare Beneficiaries With Advanced Cancer. *JAMA Health Forum*. Published February 21, 2025. doi:10.1001/jamahealthforum.2024.5436

Data
Data available: No

Additional Information
Explanation for why data not available: The datasets used to conduct this study cannot be shared directly as per the research protocol from the National Cancer Institute. Instructions for obtaining these data are available at https://healthcaredelivery.cancer.gov/seermedicare/obtain/.
